# The Over 75 Service: Continuity of Integrated Care for Older People in a United Kingdom Primary Care Setting

**DOI:** 10.5334/ijic.5457

**Published:** 2020-07-13

**Authors:** Julie MacInnes, Julia Baldwin, Jenny Billings

**Affiliations:** 1 Centre for Health Services Studies, University of Kent, Canterbury, UK; 2 Sandgate Road Medical Practice, Folkestone, UK

**Keywords:** Integrated Care, Primary Care, Frailty, Older People, Continuity of Care

## Abstract

Continuity of care is concerned with quality of care over a period of time. It describes a process by which service users and their families are co-operatively involved with health and social care professionals in managing their care needs. Continuity of care can be divided into informational, managerial and relational and has been associated with improved user- and service-related outcomes. To date, there have been few studies which examine how continuity of care is developed and maintained in integrated primary care systems. This paper explores continuity of care in an integrated Over 75 Service for people living at home with complex health and social care needs. Using a case study approach, qualitative data was collected from multiple sources including interviews with managers and professionals, users and carers, care plans, steering group minutes and field notes. Data was analysed thematically. A number of factors are identified which characterise continuity of care, namely: information sharing through direct communication between providers and the development of trusted relationships within the team; identified care co-ordinators who acted as a conduit for information and communication; the development of ongoing relationships with users and carers requiring dedicated time and accessible and flexible services delivered in the users’ own home.

## Introduction

Continuity of care has been defined and categorised in a number of ways across a variety of disciplines. In health and social care, it has been described as a hierarchical concept on three levels [1]. At the most fundamental level, *informational continuity* describes a way in which medical and social information is available to any professional caring for the user. It includes a systematic process that allows accessing and communicating this information among those involved. *Longitudinal or managerial continuity* identifies a place in the health and social care system in which the user receives most care. It enables care to occur in an accessible and familiar environment from an organised team of providers. The team is responsible for co-ordination of care, including preventative services. Haggerty et al [2] describes this as different clinicians delivering consistent and coherent management through co-ordinated and timely delivery of services. At the highest level, *Interpersonal or relational continuity* describes an ongoing relationship between a user and personal physician. The user knows the physician by name and trusts them on a personal basis. The physician assumes personal responsibility for the user’s overall health. However, relational continuity can exist with one or more clinicians [2]. Continuity of care is presented as a hierarchy in that it is suggested that lower levels need to be in place before relational continuity can occur, that is, information sharing is the most basic requirement on which care co-ordination is built. Only then can personalised, trusted relationships be developed.

Greater continuity of care is associated with improved outcomes including reduced rates of hospitalisation [3], increased adherence to medication [4], increased user satisfaction [5] and improved clinical management and preventative care [6,7]. Relational continuity in particular, is considered a cornerstone of primary care practice and describes a relationship between the General Practitioner and user that spans multiple care episodes. However, General Practitioners are under unprecedented pressure in terms of workload, difficulties with recruitment and financial constraints [8]. Coupled with this, the drive for greater efficiency in the United Kingdom (UK) and elsewhere through the provision of general practice as scale through primary care federations or networks, an extension in out-of-hours services and the use of digital technologies means it is becoming increasingly difficult to establish and maintain personal relationships. Perhaps as a result, relational continuity is reportedly low for users with complex needs, requiring high-frequency care at home [9].

A broader skill mix in primary care in which practice-based staff increasingly deliver care and provide care co-ordination is advocated in order to free up capacity [8]. Beyond the boundaries of primary care, in the UK the Five Year Forward View [10] calls for decisive steps to break down barriers to how care is provided, specifically the creation of integrated out-of-hospital care teams consisting of primary and community care, hospital specialists, mental health and social care providers. Arguably, managerial continuity of care which extends responsibility for health and social care to members of the integrated team is becoming increasingly important in the delivery of community-based care. To date, there is limited published literature on the extent to which the different dimensions of continuity of care are evident within integrated health and social care teams or how continuity of care is established and maintained. This paper explores the concept of continuity of care in relation to integrated care, for frail, older people in the United Kingdom as part of the European SUSTAIN project. SUSTAIN (Sustainable Tailored Integrated Care for Older People in Europe) was a 4-year project (2015–2019) carried out by thirteen partners from nine European countries [11]. The aim was to support and monitor improvements to established integrated care initiatives for older people living at home with multiple health and social care needs. The Over 75 Service, delivered at a primary care medical practice in the South East of England was selected as one of 14 SUSTAIN case sites delivering integrated health and social care for older people.

### Ethical considerations

Ethical approval was gained from the Research Ethics Committee REC reference 16/IEC08/0045, IRAS project ID: 216930.

## Theory and Methods

### Setting

The Over 75 Service was developed from the Enhanced Service for General Practice scheme [12], the aim of which was to proactively identify users at highest risk of unscheduled care due to the complexity of their medical needs. The service was designed on previous local work in integrated and proactive care [13]. The medical practice serves a population of approximately 12,000 patients. The Over 75 team consisted of two registered nurses (practice matrons) working in conjunction with a general practitioner and a paramedic practitioner. The wider service delivery team consisted of intermediate care practitioners, a community nurse, social workers, a mental health practitioner, a pharmacist and a representative from voluntary agencies including Age UK and a carer support group. Services provided included all medical and nursing care, including vaccinations, personal and domiciliary care, medication reviews, befriending and support and respite for carers.

Service users were identified through a computerised risk stratification tool, which identified the top 2% of users of secondary care services and those identified by the team as frail, living alone or with limited social or family support, were housebound, or were otherwise considered vulnerable. The practice matrons undertook a detailed patient assessment, including the Dalhousie Clinical Frailty Scale [14] within the users own homes, before referring to other agencies where relevant.

Communication between service providers was established via a steering group which was set up to lead the development and implementation of the service. A multidisciplinary case management approach was adopted with monthly multidisciplinary team meetings consisting of between 5–8 practitioners. A dedicated telephone number was created for users, informal carers and professionals which bypassed the busy main reception line. The practice matrons were the main co-ordinators of care, liaising between different professionals and being a single point of contact for users and carers.

### Methodology

The overall SUSTAIN methodology for all European sites employed a multiple embedded case study design [15] with data collected from each of the integrated care sites. The implementation of the integrated care initiatives and the evaluation of process and outcomes was guided by the Evidence Integration Triangle model [16], which has its origins in implementation science. The SUSTAIN methodology is described in detail in a paper by de Bruin et al. [17].

### Methods

For the purpose of this paper, data collected for the Over 75 Service as part of SUSTAIN, was analysed in order to explore the extent to which continuity of care was evident and how it was established and maintained.

### Data collection

Data consisted of qualitative interviews with users and carers; interviews and a focus group conducted with managers and professionals delivering the service; documentary analysis of care plans; minutes of steering group meetings and the researcher’s field notes (Table 1).

**Table 1:** Summary of data sources.

Data Source	(N=)

User interviews	15
Carer interviews	4
User demographic questionnaire	15
Carer demographic questionnaire	4
Care plans	15
Professionals interview	2
Professionals focus group	1 (N = 4)
Minutes of steering group meetings	16
Field notes	Continuous

Demographic data of users and carers was also collected. Data was collected between March 2017 and April 2018.

#### 1) Users and carers

The inclusion criteria for users was 75 years of age or older, living at home, with multiple health and social care needs, in receipt of the service for a minimum of 12 weeks, and cognitively able to participate in the study. Informal caregivers of users were also invited to participate. Interviews were semi-structured according to a pre-determined interview schedule, carried out face-to-face in the users or carers home and audio-recorded. The researcher completed a demographic questionnaire with the user or carer, at the time of the interview. Permission was also sought from users’ to access their care plan, which was held either in the users home or at the medical practice.

#### 2) Managers and Professionals

Managers and professionals delivering the service were invited to participate either in an interview or a focus group as determined by the availability of the participants. The interviews and focus group were semi-structured according to an interview schedule, carried out face-to-face in the medical centre and audio-recorded.

#### 3) Steering group meeting minutes and field notes

A steering group consisting of managers and professionals was set up at the start of the SUSTAIN project. Meetings took place regularly between December 2015 and April 2018. Meetings were chaired by the researcher and minuted. Reflective field notes were written by the researcher on an ongoing basis.

### Data Analysis

Qualitative data was analysed thematically using Flick’s approach [18] which involved bringing predetermined templates to the data, in this case the interview and focus group schedules. Quotes were sorted into categories and coded according to their origin. Each category was organised into themes using the quotes to justify interpretation. Any data lying outside of the predetermined themes were analysed separately and also grouped into themes. The qualitative data analysis software, NViVO11, was used to facilitate the process. Individual data sources were coded separately. Unique identification codes were assigned to users (U), carers (C), managers/professionals (M/P), care plans (CP), steering groups minutes (SG/date) and field notes (FN). Quantitative demographic data was analysed using descriptive statistics.

## Results

The demographic characteristics of users and carers are presented in Table 2.

**Table 2:** Demographic characteristics of users and carers.

User			Carer		

*Sex*	Male	5 (33.3%)	*Sex*	Male	2 (50.0%)
	Female	10 (66.7%)		Female	2 (50.0%)
*Age group*	75–84 years	7 (46.6%)	*Age group*	75–84 years	3 (750%)
	85+ years	8 (53.3%)		85+ years	1 (25.0%)
*Education level*	Low	10 (66.7%)	*Education level*	Low	2 (50.0%)
	Middle	3 (20.0%)		Middle	2 (50.0%)
	High	2 (13.3%)			
*Marital status*	Married/cohabiting	5 (33.3%)	*Marital status*	Married/cohabiting	4 (100%)
	Divorced	2 (13.3%)			
	Widowed	8 (53.3%)			
*Living situation*	Living at home alone	7 (46.7%)	*Living situation*	Living at home with user who is spouse/partner	4 (100%)
	Living at home with spouse or partner	6 (40.0%)			
	Living at home with at least one other family member	1 (6.75%)			
	Other	1 (6.7%)			
*Medical conditions*	Number of conditions	Mean = 5.20 (sd ± 2.7), (range 1–11)			

From the analysis, a number of sub-themes were identified within each domain of continuity of care (Table 3).

**Table 3:** Themes of continuity of care.

Domain	Sub-Theme

Informational	Willingness to share information
	Mechanisms for information sharing
	Personalised and family-focused care
Managerial	Greater efficiency
	An identified care co-ordinator
Relational	Trusted relationships
	Accessibility of professionals
	The value of an extended assessment

### Informational continuity

#### Willingness to share information

Overall, there was a willingness to share information across organisations and amongst different professionals although the need to share information which was not perceived to be relevant to all agencies was questioned:

“It’s kind of working together and just sharing information, rather than thinking ‘Oh we’re the district nurses and that’s the GP surgery’ and not sharing information. If we’re told something and we think it would be valuable for them to know, we’ll always pass that information over” (M/P6)

“Each organisation wants their own paperwork…also we’re all looking at slightly different things so, it’s of no interest to me how much money somebody’s got in the bank but that’s quite an inherent part of the social services assessment. So there will be different elements of different information that we want from patients that might not be relevant to other services” (M/P5)

From a user and carer perspective, information sharing was not always apparent to users and carers of the service who recognised that whilst information was shared amongst staff within the medical practice, this was not the case for outside agencies:

“I think the surgery shares things between them” (U2)

“Information is shared within the surgery [between the GPs, nurses and reception staff] but not with outside agencies [i.e other community service providers] – I always need to repeat things. I act as a sort of co-ordinator [between services]” (C1)

#### Mechanisms for information sharing

Information was shared in monthly multidisciplinary team meetings and via electronic referral systems. Importantly, more informal methods such as telephone calls and face-to-face drop-in meetings were also used as means of communication. As a result, personal relationships were established between professionals which was highly valued:

“If I’ve visited patients and have any concerns or questions I can come in at any time and ask, but it’s always a good point at the MDT [multidisciplinary team] because there’s a lot of people round the table who would also have input” (M/P4)

“Basically it’s just information sharing, it’s just a case of ringing up the surgery, speaking to them and it’s just such an easy conversation to have, you can tell them what the problem is, what you feel, and it is just a nice conversation, they’re happy to work with you” (M/P6)

The use of information technology as a facilitator of information sharing was problematic due to the lack of interoperable systems and concerns about information governance:

“The district nurses went paper-free and that caused its own issues and social services are on a completely different thing and then initially they couldn’t even send us emails because we weren’t secure enough and, you know, there’s been all these sort of problems with IT and nobody seems to have the solution” (M/P5)

“The information governance has created a few headaches…It’s knowing what you can and can’t send and share electronically or by other means” (M/P4)

From a user and carer perspective, it was believed that information stored on computer was detailed and accessible, which was in contrast to the views of the professionals:

“They seem to get everything through on the different computers” (U1)

“As far as I know they’ve got a good account of me on the computer, so I think basically there’s not much they don’t know about me really” (U10)

Care plans were not a significant method of communication between organisations, with each provider having their own care plan. As a result, care plans were used as a mechanism for sharing information with the user rather than between the team.

#### Personalised and family-focused care

Information shared across organisations was perceived to enhance care efficiency and led to more person- and family-centred care:

“Knowing the patient’s histories, means that we provide a more efficient service because there aren’t any sort of holes in what we’re doing. We can go in and see that patient and we know everything about them before we’ve even walked through their front door, that means that care can be given more efficiently because we don’t have to dig for information before going in” (M/P6)

“It’s not just the patients, the team here are very aware of the family situation and quite often they refer to me to give the husband, the wife, the son, daughter, whoever, a bit of a break because they are the main carer, so it’s knowing the whole situation at home and being able to give you that information” (M/P3)

### Managerial continuity

#### Greater efficiency

Professionals believed care was co-ordinated within the service, resulting in greater efficiency with less duplication and fragmentation of services:

“A lot of our patients are in and out of crisis and I think it’s working out how we work together because we’re obviously referring onto lots of people. It’s making sure we haven’t doubled up on everything” (M/P3)

“I think having someone to plug the gaps. I think the more we work together the more those sorts of gaps become obvious” (M/P2)

#### An identified care-coordinator

As specified in the design of the Over 75 Service, the practice matrons acted as the main co-ordinators of care, liaising between different professionals and being the single point of contact for users and carers:

“It’s easier for me when I get a referral through and they [practice matron] say, ‘right, we’ve done this, they’re going to be referred to so-and-so, they’re going to do this’” (M/P3)

Users and carers were also able to identify who was co-ordinating their care:

“I think she [practice matron] is specified as my care co-ordinator so I do feel I’ve got an open line to her” (U3)

Liaison with social service was more difficult as individual users had different case managers. This resulted in a failure to establish personal relationships between different providers and led to perceived difficulties in care co-ordination:

“One of the problems with social services is that you can’t just directly phone any individuals, you always have to go through a central point” (M/P5)

“Social Services are a bit more difficult because every patient has a different case manager…It can be slightly disjointed because you need to find out who their case manager is” (M/P6)

### Relational continuity

#### Trusted relationships

Strong relationships, particularly with the practice matrons was established with users over time.

“Being in more contact with the nurses I’ve found I know them personally now…I think it’s quite nice and they are very friendly and it’s nice to speak to them because they know who I am, and I know them” (U4)

“I trust them. You know, I mean this is the difference. The rapport is totally different with somebody that will listen to the patient than somebody that tells you what you’ve got to do” (U7)

In contrast, there was some lack of relational continuity with social services carers:

“If I get the regular one she’s knows more or less anyway but sometimes we have a different one come in” (C4)

#### Accessibility of professionals

The availability and responsiveness of the practice matrons were particularly highly valued:

“She’s given me a telephone number so I can get in touch if I want any help” (U10)

“You’ve only got to ring up the surgery and she’s here in about 3 minutes if you really need her. She’s always here if I badly need her” (U7)

The practice matrons would also ‘pop-in’ which added to the sense that users and carers were valued:

“One of the nurses did call and see me a few weeks ago and she said she was just in the area and just popped in to see if I was alright” (U4)

“[User] had bronchitis pretty badly and then the matron came back and sat and had a lovely talk to us about all sorts of relevant things which is helpful and just to check up on [user]” (C2)

#### The value of an extended assessment

The extended length of time the practice matrons were able to spend with users and carers on an initial assessment, in their own home was instrumental in developing relationships:

“I had a very useful talk with [practice matron]. I had to have an extended talk with her, it was very good… we did have a good old chat then, but that’s really the first time in all my experience of the NHS [National Health Service]” (U3)

“She asked me what I’d had wrong with me, why I was in this state that I am… just asked me what illnesses I’d had recently but just general conversation about things. She was very, very pleasant and very…you felt you could talk to her. She wasn’t in a rush” (U11)

This however contrasted with the limited time spent with a general practitioner, where due to time pressures, users are given specific time slots, with resulting perceived lack of continuity:

“You are conscious of that time factor, that it’s 10 minutes and that’s your lot really” (U9)

“You can have a 10 minute appointment with any one of the doctors…sometimes they suddenly get up as much as to say ‘well it’s time you went now’ (U11)

## Discussion

The continuity of care hierarchy consists of some of the main tenets of integrated care working namely, information sharing, care co-ordination and developing and maintaining interpersonal relationships between professionals and with users and carers and so is a useful lens through which to explore integrated care. In this analysis, an examination of continuity of care provides insights into primary care as a setting for integrated care, multidisciplinary teamworking and the transferability and sustainability of integrated care initiatives. A high degree of continuity of care, as found in the Over 75 Service, indicates a highly integrated network [19].

### Primary care as an integrated care setting

In this study, a primary care setting facilitated integration as the medical practice acted as an informational and geographical hub, providing a focal point for community service providers, users and carers. Unlike other services, general practice records care episodes over long periods of time, which provides rich and comprehensive information about a user which has the potential to be shared. In the Over 75 Service, the responsibility for care co-ordination lay with practice matrons rather than general practitioners. This is in line with the vision of healthcare in the United Kingdom where practice-based staff, in this case nurses, provide care co-ordination in order to free up capacity amongst general practitioners [8]. The practice matrons fulfilled a dual role in establishing and maintaining both managerial and relational continuity. They were the central point of contact for members of the integrated care team and were also the main point of contact for users and carers accessing services across multiple care episodes. The senior practice matron was well-positioned to lead the service given their engagement with users and carers and the presence of existing relationships with other professionals. As a consequence, managers and professionals in the team perceived a high degree of managerial continuity where different professionals delivered consistent and coherent management through co-ordinated and timely delivery of services [2]. This resulted in perceived reduced duplication of services, fewer gaps in service provision and greater efficiency through more focused interventions. Importantly, unlike the general practitioners, the practice matrons had dedicated time to deliver the Over 75 Service which was crucial in terms of both establishing and maintaining the initiative.

The way in which provider organisations were structured around the primary care medical practice, impacted on managerial continuity. Individual staff members from different provider organisations were assigned to the practice so each covered the same geographical footprint with responsibility for the same group of service users. As a result, the team was relatively small and had frequent opportunities to work together and became known to each other personally. However, this was not the case for social services which was not geographically aligned to the primary care practice but rather, was part of a region-wide, highly centralised service. This meant that different case managers, accessed via an automated referral system, were assigned to different users leading to a failure to establish personal relationships with other members of the team and perceived difficulties in care co-ordination. This was also evident for users where co-ordination for social care was seen as the responsibility of social services, rather than the practice matrons as for all other services. The lack of a standardised information system has been cited as a barrier to integrated primary care services for older people [20].

Relational continuity was established primarily with the practice matrons. In a recent review of integrated care for older people with frailty, it was found that service users and carers placed importance on continuity of care through one-to-one relationships with a care co-ordinator, valuing this relationship to provide information and support and facilitate personalised care [21]. The main facilitators and maintenance factors for relational continuity, in this study, where the accessibility of the matrons via a direct telephone line and their ability to respond quickly when needed. This is consistent with published literature where having a single trusted professional who helps navigate the system and sees the patient as an equal partner supports the experience of continuity of care for users [2]. Equality of the relationship between older people and their case manager has been described as condition for achieving productive interactions [22].

However, this continuity may have highlighted the lack of a relationship with the general practitioner where users rarely saw the same practitioner and only for time-limited consultations. However, perceived effective information sharing is known to compensate for inconsistency in personnel and lack of relational continuity [23].

### Multidisciplinary teamworking

Within the multidisciplinary team, information exchange was key to providing care which was co-ordinated and seamless. A willingness to trust other team members and share information across organisational and professional boundaries was important in the establishment of the Over 75 Service. Establishing mutual trust between team members has been cited as a pre-requisite for integrated primary care teams caring for older people [24].

However, the mechanisms by which information was shared was also important for building trusting relationships between team members and facilitating shared decision-making and a problem-solving approach to care. Direct communication between individuals through multidisciplinary team meetings, informal meetings and telephone calls was the main vehicle for information exchange. Rather than enhancing the flow of information, information technology acted as a barrier to communication due to multiple platforms which were not interoperable and concerns about information governance where there was a lack of understanding about what information could and could not be shared digitally. This lack of interoperable information technology systems has been well-documented in integrated care [25,26]. The lack of confidence in informational technology may have stimulated face-to-face and telephone information exchange which strengthened interpersonal relationships and enhanced teamworking. In contrast, there was an assumption amongst users and carers that information was shared digitally, especially amongst primary care practitioners but less so with other community agencies. This is consistent with documented findings [2] where information sharing between professionals was assumed by users, until proven otherwise.

In the Over 75 Service, care plans were not a significant method of communication between organisations. The decision not to share care plans or to develop a shared, common document was made during the development of the service on the basis that information would not be relevant to all providers and perceived difficulties with changing organisation-wide templates. The decision whether or not to share their care plans with different providers was, therefore, left to the users. Perhaps the lack of relevance of the care plan to different organisations meant that the purpose and value of the care plans was not obvious to users. Other studies [2,11] also found that care plans were rarely seen as an aid to continuity by users. This lack of the use of care plans as an aid to continuity may be a lost opportunity to enhance care co-ordination and managerial continuity as well as to engage users in decision-making about their care.

Within the multidisciplinary team, trusted relationships with a small number of key professionals, has been found to be important in integrated teams [27]. The size of the team is therefore important in delivering integrated care. In the UK, the move to deliver primary care at-scale through primary care networks (PCNs) serving populations of 30,000–50,000 people [28] is likely to result in larger teams which may have consequences for care coordination and the development of trusting relationships.

Users and carers valued the opportunity to discuss a wide range of issues and concerns which was made possible by extended appointments with the practice matrons in particular, but also with other professionals. Care was delivered, by all professionals and voluntary care workers, in the users own home which was highly valued by both users and carers. Home visits were conducted as part of initial assessments by the practice matrons, in response to a crisis or on a more impromptu basis. A comprehensive initial assessment, has been judged of particular importance in other studies on integrated primary care for older people [29]. This care setting was important in establishing and maintaining relational continuity both from a user and professional perspective as it was felt that the environment led to the relationship being less hierarchical and medically-dominated. This emphasis on the delivery of flexible services delivered when necessary, especially where support can be provided in the person’s home, has been described as being important for continuity of care [30].

The concept of continuity of care as a hierarchy assumes information sharing is necessary for effective care co-ordination and are pre-requisites for the development of trusted relationships. From a professional perspective, the act of sharing information and working closely as a team to co-ordinate care does seem to lead to the development of close, personal relationships. However, a degree of trust is necessary before professionals are willing to share information, suggesting an inversion of this hierarchy. Lessons from the SUSTAIN project, amongst other literature [24], also suggest that the implementation of integrated care initiatives are facilitated when teams have a past history of working together and so have had time to develop good working relationships. From a user perspective, relational continuity exists independently of whether professionals share information or work together as it is based upon personal relationships with individual professionals. Continuity of care as a hierarchy therefore, depends upon whose perspective it is viewed from and may not be as linear as predicted.

### Implications for transferability and sustainability

Locating an integrated care initiative within a primary care setting had significant benefits in terms of information sharing, care co-ordination and relational continuity. Within the region, where most services are configured to align with primary care medical practices, the Over 75 Service model of care has the potential to be highly transferable across the primary care sector. The need for primary care to be better integrated into home and community care is echoed elsewhere [31]. However, the degree to which the service is sustainable is less clear. The practice matrons had dedicated funding to undertake both the leadership and delivery of the service. The voluntary services, in particular, were vulnerable to sudden discontinuation of funding. Sustainability is therefore, highly dependent on continued funding. Leadership of the service was dependent on one key individual, the senior practice matron, who had established relationships with the other members of the multidisciplinary team and users and carers over a relatively long period of time. Whilst interpersonal skills, motivation and commitment are not of course, unique to this individual, their skill and experience in leading the service means they would be difficult to replace. This over-reliance on one individual is a considerable risk to the sustainability of the service.

### Recommendations

- Integrated care initiatives are ideally located within primary can as, unlike other health and social care settings, general practice records care episodes over long periods of time. As a result, they act as ‘informational hubs’, providing a focal point for community service providers, users and carers.
- A designated care co-ordinator should be identified who is the main point of contact for service providers, users and carers. Dedicated time should be given to this role.
- Multidisciplinary teams should be small, delivering services across the same geographical footprint so that there are numerous opportunities for team members to interact and develop trusting relationships.
- Service providers should be accessible to users, and provide a flexible and responsive service as this facilitates relational continuity.

### Limitations of the study

This study is limited in that it is a single case study with a small sample size conducted as a secondary analysis from the SUSTAIN data. As a result, data was not collected specifically to explore continuity of care, although information sharing, care-coordination and person-centredness were key themes within SUSTAIN. The relationship between continuity of care and user- and service-related outcomes has not been explored in this study.

## Conclusion

The Over 75 Service presents an effective model of integrated care for older people with complex needs living at home. A primary care setting for integrated community services provides an informational and geographical hub, which acts as a focal point for health and social care providers, users and carers. Organisational structures in which individual practitioners are aligned to the same geographical footprint as the primary care practice is important for building relationships between team members and providing continuity of care for users and carers. The existence of named care co-ordinators, based in primary care who are responsible for organising care and referring to other care providers also facilitates integration. However, an over-reliance on a single individual in a leadership role threatens sustainability. Key to multidisciplinary teamworking and care co-ordination is a willingness to share information and the establishment of close, trusted relationships which is made possible by having a relatively small team with a common caseload which provides frequent opportunities for interaction and joint working. The existence of these factors in integrated primary care settings is, therefore, likely to lead to better continuity of care.
